# COMP report: CPQR technical quality control guidelines for kilovoltage X ray radiotherapy machines

**DOI:** 10.1002/acm2.12228

**Published:** 2017-11-22

**Authors:** Christophe Furstoss

**Affiliations:** ^1^ Département de Radio‐Oncologie Hôpital Maisonneuve‐Rosemont Montréal QC Canada

**Keywords:** kilovoltage, quality control, radiotherapy

## Abstract

The Canadian Organization of Medical Physicists (COMP) in close partnership with the Canadian Partnership for Quality Radiotherapy (CPQR) has developed a series of Technical Quality Control (TQC) guidelines for radiation treatment equipment. These guidelines outline the performance objectives that equipment should meet in order to ensure an acceptable level of radiation treatment quality. The TQC guidelines have been rigorously reviewed and field tested at various Canadian radiation treatment facilities. The development process enables rapid review and update to keep the guidelines current with changes in technology (the most updated version of this guideline can be found on the CPQR website). This particular TQC details recommended quality control for kilovoltage X Ray radiotherapy machines.

## 1 INTRODUCTION

The Canadian Partnership for Quality Radiotherapy (CPQR) is an alliance among the three key national professional organizations involved in the delivery of radiation treatment in Canada: the Canadian Association of Radiation Oncology (CARO), the Canadian Organization of Medical Physicists (COMP), and the Canadian Association of Medical Radiation Technologists (CAMRT). Financial and strategic backing is provided by the federal government through the Canadian Partnership Against Cancer (CPAC), a national resource for advancing cancer prevention and treatment. The mandate of the CPQR is to support the universal availability of high‐quality and safe radiotherapy for all Canadians through system performance improvement and the development of consensus‐based guidelines and indicators to aid in radiation treatment program development and evaluation.

This document contains detailed performance objectives and safety criteria for Kilovoltage X Ray Radiotherapy Machines. Please refer to the overarching document Technical Quality Control Guidelines for Canadian Radiation Treatment Centres^1^ for a programmatic overview of technical quality control, and a description of how the performance objectives and criteria listed in this document should be interpreted.

All information contained in this document is intended to be used at the discretion of each individual center to help guide quality and safety program improvement. There are no legal standards supporting this document; specific federal or provincial regulations and license conditions take precedence over the content of this document.

## 2 SYSTEM DESCRIPTION

Kilovoltage radiotherapy units, although eclipsed first by ⁶⁰Co irradiators and then by linear accelerators, remain useful in the mix of energies available to a radiotherapy program. Low‐energy X ray beams have application in the treatment of skin lesions and shallow tumors. The quality assurance program for the kilovoltage units must match the rigor of that for the most modern irradiators and is every bit as important in order to safely deliver an accurate dose to the patient for those lesions they are appropriate to treat.

Application of kilovoltage radiotherapy is divided into two categories based on the chosen tube voltage. The lower energy range (the “superficial” range; X ray tube potentials of 30 kVp or 40 kVp to 100 kVp and tube currents of a few milliamperes) is used to treat surface lesions. Filtration of up to 6 mm of Al is added to remove the very low energy photons and “harden the beam.” Applicator cones, attached directly to the tube‐housing head, are the usual method by which the irradiation area is defined. Variable collimators are also available on some units and require additional quality control tasks over those performed for applicators. Treatment is performed at short source‐to‐surface distance (SSD) (e.g., less than 20 cm) and the lesion depth must be less than a few millimeters. Therefore, the “kilovoltage” range is selected when surface to shallow lesions are treated. In so doing, tissue greater than that at a moderate depth is spared when treating surface lesions.

“Orthovoltage” therapy refers to radiation treatment using X ray tube potentials in the 100–300 kVp range, although 200−300 kVp may be the more practical specification. This deeper radiotherapy equipment uses beam currents of up to 20 mA and applied filtration equivalent to produce half‐value layer (HVL) values of 0.1–4 mm Cu. Coned applicators or movable diaphragms are used to define these beams. While coned applicators may be constructed mostly of metal (e.g., Cu), they have a clear plastic end to aid in viewing the target region. Hence, attention must be given to the integrity of the plastic portion. The depth dose distribution in the orthovoltage energy range is dependent on factors such as kVp, HVL, SSD, and field size. Maximum dose occurs close to the skin, with 90% of the dose being delivered within a tissue depth (water depth) of 2 cm.

Detailed descriptions of various types of kilovoltage X ray radiotherapy machines and various quality control tests have been published in the literature.^2^, ^3^, ^4^, ^5^, ^6^, ^7^, ^8^, ^9^, ^10^, ^11^, ^12^, ^13^, ^14^, ^15^, ^16^, ^17^, ^18^, ^19^, ^20^, ^21^, ^22^, ^23^, ^24^, ^25^, ^26^

## 3 RELATED TECHNICAL QUALITY CONTROL GUIDELINES

In order to comprehensively assess kilovoltage X ray radiotherapy machines performance, additional guideline tests as outlined in related CPQR Technical Quality Control (TQC) guidelines must also be completed and documented, as applicable. Related TQC guidelines, available at cpqr.ca, include:

1. Safety Systems
2. Major Dosimetry Equipment

## 4 TEST TABLES (TABLES 1, 2, 3, 4)

**Table 1:** Daily quality control tests

Designator	Test	Performance	
		Tolerance	Action
Daily			
DK1	kVp and mA indicators	Functional	
DK2	Beam‐off at key‐off test	Functional	
DK3	Beam interrupt	Functional	
DK4	Backup timer/monitor unit channel check	1%	2%
DK5	Dosimetric test: output check	2%	4%

**Table:** Notes on daily tests

DK1	Functional check of kVp and mA indicators
DK2	Functional check of beam‐off at key‐off
DK3	Functional check of the beam interrupt button (radiation stop and restart when the radiation on button is pressed again)
DK4	Quantitative verification of correct operation of backup timer or backup monitor unit
DK5	Quantitative dosimetric test: output reproducibility test at the chosen energies and filter combinations. If the output is stable with time, this test can be done weekly (so replaced by WK4 according to Table 2) on the condition that there are documents and reports to support it

**Table 2:** Weekly quality control tests

Designator	Test	Performance	
		Tolerance	Action
Weekly			
WK1	Couch movement and brakes	Functional	
WK2	Unit motions and motion stops	Functional	
WK3	Interlocks for added filters/kVp‐filter choice	Functional	
WK4	Dosimetric test: output check	2%	4%

**Table:** Notes on weekly tests

WK1	Functional check of couch motion and brakes (where applicable)
WK2	Functional check of unit motions and motion stops
WK3	Functional check of interlocks for added filters, correct placement of filters, and the matching of filters with kVp value
WK4	Quantitative dosimetric test: output reproducibility test for all energies. This test can be limited to the energies used clinically on the condition that any additional energies cannot be chosen at any time (by removing the corresponding filters from the treatment room, for example)

**Table 3:** Monthly quality control tests

Designator	Test	Performance	
		Tolerance	Action
Monthly			
MK1	Mechanical stability and safety	Functional	
MK2	Cone/filters integrity and cone indicators	Functional	
MK3	Physical distance indicators	2 mm	3 mm
MK4	Accuracy of head tilt and rotation readouts	1°	1.5°
MK5	Light/x ray field coincidence	2 mm	3 mm
MK6	Light field size	2 mm	3 mm
MK7	X ray field size indicator	2 mm	3 mm
MK8	X ray field uniformity	5%	8%
MK9	Output verification and reproducibility with head tilt and rotation	2%	4%
MK10	Timer accuracy verification	1%	2%
MK11	Dose rate output constancy	2%	4%
MK12	Records	Complete	

**Table:** Notes on monthly tests

MK1	Verification that the unit and accessories are firmly anchored and may be used without endangering patients or staff
MK2	Verification of the integrity of the filters and cones and cone indicators
MK3	Verification of the optical and/or mechanical distance indicator if the unit is equipped with one
MK4	Verification of the angle readouts
MK5	Performance parameters refer to agreement at each edge. This test does not apply to all machine designs
MK6	Geometric test to verify the light field sizes (where applicable)
MK7	Confirmation of radiation field size when a variable collimation system is provided. At least two field sizes must be checked
MK8	Using a film, the flatness and symmetry of the X ray beam must be assessed for the largest cone
MK9	Quantitative dosimetric test: output reproducibility test at all energies with varying head tilt and rotation^27^
MK10	If the unit is equipped with a timer, its accuracy must be checked against a stop watch over a range of doses of 10−1000 cGy
MK11	Should be checked for all beam qualities for MU‐based systems
MK12	Documentation relating to the daily quality control checks, preventive maintenance, service calls and subsequent checks must be complete, legible, and the operator identified

**Table 4:** Annual quality control tests

Designator	Test	Performance	
		Tolerance	Action
Annual			
AK1	Reference dosimetry	1%	2%
AK2	Timer and end‐effect error	Characterize	±0.05 min
AK3	Output linearity	n/a	1%
AK4	Output reproducibility	2%	3%
AK5	Output error associated with beam interrupt	2%	4%
AK6	Beam quality	10%	15%
AK7	Alignment of focal spots	0.5 mm	1 mm
AK8	Focal spot size	Reproducible	
AK9	Percentage depth dose verification	Characterize and document	
AK10	Profiles verification	Characterize and document	
AK11	Independent quality control review	Complete	

**Table:** Notes on annual tests

AK1	Using a high‐quality dosimetry system calibrated against the local secondary standard, all beams and cones in use are recalibrated
AK2	Timer and end‐effect error measurement may be performed in conjunction with AK3
AK3	Output linearity measurement for a clinically used filter/cone combination at a standard SSD and a dose range of 10−1000 cGy
AK4	Output reproducibility verification for a clinically used filter/cone combination. These measurements should be repeated at typical tilt and head rotation used for treatments
AK5	Output error when the beam is interrupted during the irradiation for a clinically used filter/cone combination
AK6	The HVL of any clinically used beams is measured. The HVLs measured in millimeters of Al or millimeters of Cu as appropriate are compared with the values obtained at commissioning. These tolerances acknowledge measurement uncertainty
AK7	Focal spot—quantitative measurement, assessed relative to acceptance test value where applicable
AK8	Using a pin hole or resolution tool
AK9	Verification of percentage depth dose measurements for all used filter/cone combinations against baseline
AK10	Verification of inplane and crossplane profiles at different depths for all used filter/cone combinations against baseline
AK11	To ensure redundancy and adequate monitoring, a second qualified medical physicist must independently verify the implementation, analysis, and interpretation of the quality control tests at least annually

## CONFLICT OF INTEREST

No conflict of interest.
